# Elevated Serum Tsukushi Levels in Patients With Hyperthyroidism

**DOI:** 10.3389/fendo.2020.580097

**Published:** 2020-09-29

**Authors:** Deying Liu, Peizhen Zhang, Xueyun Wei, Yajuan Deng, Wenhui Liu, Dan Guo, Jianfang Liu, Bingyan Xu, Chensihan Huang, Junlin Huang, Jiayang Lin, Shiqun Liu, Yaoming Xue, Huijie Zhang

**Affiliations:** Department of Endocrinology and Metabolism, Nanfang Hospital, Southern Medical University, Guangzhou, China

**Keywords:** Tsukushi, hyperthyroidism, thyroid hormone, metabolism, hepatokine

## Abstract

**Background:** Tsukushi (TSK) is a secreted hepatokine recently identified as playing an important role in modulating glucose and lipid metabolism, and systemic energy homeostasis. However, information is not available regarding the association between circulating TSK and hyperthyroidism in humans.

**Methods:** We measured serum TSK levels in 180 patients with hyperthyroidism and 82 healthy controls recruited from the clinic. Of them, 46 hyperthyroid patients received thionamide treatment for 3 months.

**Results:** Hyperthyroid patients had higher levels of circulating TSK than healthy controls [186.67 (133.63–280.59) ng/ml vs. 97.27 (77.87–146.96) ng/ml, *P* < 0.001]. Subjects with higher level of serum free triiodothyronine (T3) and free thyroxine (T4) had higher levels of circulating TSK. In addition, serum TSK levels markedly declined with the improvement of thyroid function after thionamide treatment. In multivariable linear regression analyses, circulating TSK concentrations were significantly associated with serum free T3, free T4, thyroid stimulating hormone, thyrotropin receptor antibody, total cholesterol, low-density lipoprotein cholesterol (LDL-cholesterol), high-density lipoprotein cholesterol (HDL-cholesterol), and basal metabolic rate (all *P* < 0.01), adjusting for age, gender, smoking, and body mass index (BMI). Importantly, circulating TSK was significantly associated with risks of hyperthyroidism in multivariable logistic regression analyses, adjusting for age, gender, smoking, BMI, fasting glucose, LDL-cholesterol, and insulin resistance (HOMA-IR) [OR (95% CI), 1.012(1.005–1.019), *P* = 0.001].

**Conclusion:** These findings indicate that circulating TSK concentrations are independently associated with hyperthyroidism, suggesting that circulating TSK may be a predictive factor of hyperthyroidism and can be used for therapeutic monitoring.

## Introduction

Hyperthyroidism is a condition that occurs due to excessive thyroid hormone synthesis and secretion from the thyroid gland. Hyperthyroidism is characterized by hypermetabolic state, such as weight loss, increased energy expenditure, accelerated lipolysis and hepatic gluconeogenesis (1). Graves' disease remains the most common cause of hyperthyroidism in iodine-sufficient areas (1). It has been well-documented that excess thyroid hormone promotes metabolic processes through its two receptor isoforms, thyroid hormone receptor α (TRα) and thyroid hormone receptor β (TRβ) (2, 3). Thyroid hormone modulates thermogenesis through TRα that is mainly expressed in the skeletal muscle, adipose tissue and heart, whereas it regulates lipid metabolism through TRβ in the liver (3–5). Studies have shown that thyroid hormone signals were involved in crosstalk with metabolic signaling pathways, such as β-adrenergic signaling and uncoupling protein (UCP)1-dependent thermogenesis in white adipose tissue and skeletal muscle, and peroxisome proliferator-activated receptor (PPAR) α and liver X receptor α pathway in liver (6–9).

Recent advances suggest that the liver may impact metabolism through the secretion of hepatokines, which mediate interorgan communication through autocrine, paracrine, and endocrine signaling (10, 11). Several hepatokines including fetuin A, fibroblast growth factor 21 (FGF21), retinol-binding protein 4 (RBP4), and neuregulin 4 (Nrg4) (12–15), have been identified to involve in the thyroid hormone signaling network to modulate whole-body glucose and lipid homeostasis. These observations underscore the importance of hepatokines in crosstalk with thyroid hormone signaling pathway in pathogenesis of hyperthyroidism.

Recently, Tsukushi (TSK) has been identified as playing an important role in modulating organ developmental processes, whole body energy expenditure and metabolic homeostasis (16–19). Wang et al. have identified TSK as a hepatokine that is tightly linked to the activation of thermogenesis and energy expenditure (20). Previous studies have shown that TSK impacts systemic cholesterol homeostasis by lowering circulating high-density lipoprotein cholesterol and cholesterol efflux capacity, and acts as a blood biomarker of liver stress linking non-alcoholic steatohepatitis (NASH) and the development of atherogenic dyslipidemia and atherosclerosis (21, 22). Evidence indicated that hepatic TSK expression and secretion were strongly induced by stimuli which increased thermogenesis and energy expenditure, such as thyroid hormone, adrenergic agonist and cold exposure and TSK was identified as a negative regulator of sympathetic innervation in brown fat, and suppressed thyroid hormone expression and catecholamine synthesis (20).

Despite there are observations on metabolic crosstalk of TSK with thyroid hormone signal in animal models, information is not available regarding the association between circulating TSK concentrations and hyperthyroidism in humans. In this study, we aimed to investigate the change of TSK levels in patients with hyperthyroidism during thionamide treatment and explore the associations of circulating TSK with thyroid function and metabolic risk factors in patients with hyperthyroidism.

## Subjects and Methods

### Study Participants

A total of 180 participants with Graves' disease in Nanfang Hospital of Southern Medical University, Guangzhou, China, were recruited from August 2017 to March 2020. Graves' disease was diagnosed by typical clinical presentation, elevated serum thyroid hormone, reduced thyroid stimulating hormone (TSH), and elevated serum TSH receptor antibody (TRAb) levels. Eighty-two healthy controls were recruited from physical examination center of Nanfang Hospital of Southern Medical University. Of these, 46 patients with hyperthyroidism received thionamide treatment for 3 months, and follow-up visit occurred when their thyroid hormone levels reached normal range. All subjects completed a physical examination and a standard questionnaire including social-demographic status, lifestyle habits, and medical history. Subjects were excluded if they had cancer, treatment with antithyroid drugs or systemic corticosteroids before enrollment, pregnancy, lactation, subacute thyroiditis, abnormal kidney function, and infectious diseases.

All subjects provided written informed consent. The study protocol was approved by the Institutional Review Board of Nanfang Hospital of Southern Medical University. The methods were carried out in accordance with the approved guidelines.

### Clinical and Biochemical Measurements

Anthropometric measurements included height, weight, waist circumference, heart rate, and blood pressure (BP). Body mass index (BMI) was calculated as weight in kilograms divided by the square of the height in meters. Waist circumference was measured at the level of the umbilicus. Three measurements were obtained with a non-stretchable tape, and the mean value was used for analysis. BP was assessed in triplicate using an electronic sphygmomanometer (OMRON Company). Basal metabolic rate (BMR) was estimated by Gale's formula: BMR = pulse pressure difference + pulse rate – 111. The mean values of the three readings were used for analysis.

Subjects were instructed to fast for 12 h before screening. Triglycerides (TG), total cholesterol, and high-density lipoprotein cholesterol (HDL-cholesterol) were measured by enzymatic colorimetric methods with automatic multichannel chemical analyzer (Hitachi 7450, Tokyo, Japan). Low-density lipoprotein cholesterol (LDL-cholesterol) was calculated by Friedewald's formula: LDL-cholesterol (mmol/L) = total cholesterol (mmol/L) – HDL-cholesterol (mmol/L) – TG (mmol/L)/2.2. Serum alanine aminotransferase (ALT) and aspartate aminotransferase (AST) were measured by standard enzymatic methods. Serum gamma-glutamyltransferase (GGT) was measured by the Szasz-Persijn method. Fasting plasma glucose concentrations were measured using the glucose oxidase method. Serum insulin, free triiodothyronine (T3), free thyroxine (T4), and thyroid stimulating hormone (TSH), thyroid peroxide antibody (TPOAb), thyroglobulin antibody (TGAb), and thyrotropin receptor antibody (TRAb) concentrations were measured using electrochemiluminescence immunoassay (Roche Diagnostics). Insulin resistance status was assessed using the homeostasis model assessment of insulin resistance (HOMA-IR) according to the following formula: HOMA-IR = fasting serum insulin (μU/mL) × fasting plasma glucose (mmol/L)/22.5.

### TSK Measurement

Serum TSK concentrations were measured using ELISA kit (ELH-TSKU; RayBiotech, Inc.) according to the manufacturer's instruction. The assay has been proven to be highly sensitive to human TSK. The minimum detectable dose of Human TSK was determined to be 0.63 ng/ml. The intra- and inter-assay coefficients of variation were <10% and <12%, respectively.

### Statistical Analysis

All statistical analyses were performed with SAS version 9.3 (SAS Institute, Cary, NC). Data are presented as means ± standard deviation (S.D) or median (interquartile range). Data that were not normally distributed were logarithmically transformed before analysis. The subjects were classified into hyperthyroid subjects and healthy controls or four quartiles according to serum TSK levels by gender in subjects with hyperthyroid. The χ^2^-test and logistic regression models were used for comparison of categorical variables between groups. Analyses of covariance were performed using general linear models (GLM) to test differences in study variables between two groups or different quartiles of serum TSK levels. The correlation of serum TSK levels with multiply risk factors was analyzed by Pearson correlation coefficients. Multivariable logistic regression models were used to examine the association of serum TSK levels with risks of hyperthyroidism, adjusted for age, gender, smoking, BMI, fasting glucose, HOMA-IR, and LDL-cholesterol. Two-sided values of *P* < 0.05 were considered statistically significant.

## Results

### Circulating TSK Levels in Patients With Hyperthyroidism

Table 1 summarizes the mean levels of study variables in subjects with hyperthyroidism and healthy controls. The mean age of the hyperthyroid patients was 33.5 ± 10.5 years. Compared with healthy controls, hyperthyroid patients had higher levels of serum thyroid hormones and thyroid antibodies, including free T3, free T4, TRAb, and TPOAb, and lower levels of serum TSH. Additionally, hyperthyroid patients had higher levels of BMR, heart rate, ALT, AST and serum triglyceride, and lower levels of diastolic BP, total cholesterol, LDL-cholesterol, and HDL-cholesterol than the healthy controls. There was no difference in BMI, waist circumference, fasting glucose, HOMA-IR, or serum TGAb levels between the two groups. Of interest, hyperthyroid patients had higher levels of circulating TSK than the controls [186.67 (133.63–280.59) ng/ml vs. 97.27 (77.87–146.96) ng/ml; *P* < 0.001].

**Table 1:** Clinical characteristics of hyperthyroid patients and control.

**Variables**	**Controls**	**Hyperthyroid Patients**	***P*-value^§^**
Sample size	82	180	–
Age (years)	30.9 ± 9.2	33.5 ± 10.5	0.052
Gender (female *n*, %)	59 (72.0)	125 (69.4)	0.682
Current smokers (*n*, %)	0 (0)	12 (6.7)	**0.021**
BMI (kg/m^2^)	21.1 ± 2.7	20.5 ± 3.2	0.051
Waist circumference (cm)	74.4 ± 9.0	74.7 ± 8.4	0.895
Systolic BP (mmHg)	119.4 ± 12.0	122.6 ± 15.3	0.180
Diastolic BP (mmHg)	74.8 ± 7.8	71.8 ± 10.8	**0.013**
Heart Rate (beats/minute)	77.9 ± 8.4	104.8 ± 17.2	**<0.001**
Triglycerides (mmol/L)^a^	0.74 (0.61–0.97)	0.88 (0.70–1.11)	**0.030**
Total cholesterol (mmol/L)	4.77 ± 0.79	3.59 ± 0.81	**<0.001**
LDL-cholesterol (mmol/L)	2.98 ± 0.69	1.87 ± 0.61	**<0.001**
HDL-cholesterol (mmol/L)	1.40 ± 0.30	1.26 ± 0.33	**0.003**
Fasting glucose (mmol/L)	4.97 ± 0.60	5.13 ± 0.64	0.203
HOMA-IR^a^	1.79 (1.23–2.36)	1.37 (0.96–2.16)	0.052
ALT (U/L)	17.8 ± 12.2	35.7 ± 14.3	**<0.001**
AST (U/L)	21.1 ± 5.3	27.4 ± 10.4	**<0.001**
Free T3 (pmol/L)^a^	4.99 (4.65–5.42)	26.20 (15.10–89.16)	**<0.001**
Free T4 (pmol/L)^a^	14.98 (13.82–16.41)	63.90 (38.68–83.47)	**<0.001**
TSH (mIU/L)^a^	1.83 (1.26–2.85)	0.01 (0.00–0.01)	**<0.001**
TPOAb (IU/ml)^a^	6.85 (3.27–11.99)	26.38 (5.90–192.50)	**<0.001**
TGAb (IU/mL)^a^	12.84 (8.38–21.48)	26.04 (11.46–243.91)	0.927
TRAb (IU/L)^a^	0.30 (0.30–0.30)	17.19 (7.21–29.81)	**<0.001**
BMR (%)	11.50 ± 10.94	44.62 ± 22.32	**<0.001**
Serum Tsukushi (ng/ml)^a^	97.27 (77.87–146.96)	186.67 (133.63–280.59)	**<0.001**

*BMI, body mass index; BP, blood pressure; LDL, low density lipoprotein; HDL, high density lipoprotein; HOMA-IR, homeostasis model assessment of insulin resistance; ALT, alanine transaminase; AST, aspartate aminotransferase; T3, triiodothyronine; T4, thyroxine; TSH, thyroid stimulating hormone; TPOAb, thyroid peroxide antibody; TGAb, thyroglobulin antibody; TRAb, thyrotropin receptor antibody; BMR, basal metabolic rate*.

*Data are presented as the mean ± SD or median (interquartile range)*.

§ *Adjusted for age and gender*.

a *Analysis performed on log-transformed data. Two-sided values of P <0.05 were considered statistically significant*.

### Association of Circulating TSK Level With Metabolic Risk Factors and Thyroid Hormones

Table 2 presents clinical characteristic by quartiles of serum TSK levels in hyperthyroid subjects, adjusted for age and gender. There were no differences in BMI, systolic BP, diastolic BP, fasting glucose, triglycerides, LDL-cholesterol, HDL-cholesterol, HOMA-IR, TSH, and TPOAb among the four quartiles of serum TSK levels. Compared to subjects in the lowest quartile of serum TSK levels, those in the highest quartile had higher levels of BMR, heart rate, TGAb, and TRAb, and lower levels of total cholesterol (all *P* < 0.05). Furthermore, free T3 and T4 gradually increased across increasing quartiles of serum TSK levels (*P* < 0.001 and *P* = 0.011, respectively).

**Table 2:** Clinical characteristics by quartiles of serum Tsukushi levels in hyperthyroid subjects.

**Variables**	**Serum Tsukushi level**				***P*-value for trend^§^**
	**Quartile 1**	**Quartile 2**	**Quartile 3**	**Quartile 4**	
Sample size	44	45	46	45	
Serum Tsukushi (ng/ml)^a^	105.29 (84.85–123.77)	157.20 (143.81–166.15)^‡^	219.48 (201.93–256.79)^‡^	391.49 (349.20–452.07)^‡^	**<0.001**
Age (years)	34.2 ± 11.0	31.9 ± 10.4	34.1 ± 10.0	33.9 ± 10.6	0.705
Gender (female *n*, %)	31 (70.5)	31 (68.9)	32 (69.6)	31 (68.9)	0.998
BMI (kg/m^2^)	20.4 ± 2.6	21.1 ± 4.4	20.6 ± 2.7	19.8 ± 2.6	0.213
Systolic BP (mmHg)	120.7 ± 13.5	122.1 ± 17.9	122.8 ± 14.8	124.6 ± 15.0	0.683
Diastolic BP (mmHg)	71.8 ± 10.6	71.6 ± 10.5	72.8 ± 10.9	70.9 ± 11.5	0.883
Heart rate (beats/minute)	100.4 ± 16.4	102.1 ± 16.1	105.2 ± 20.1	111.2 ± 14.1^‡^	**0.005**
BMR (%)	38.21 ± 19.30	41.98 ± 24.32	44.14 ± 23.20	53.86 ± 19.60^‡^	**0.006**
Triglycerides (mmol/L)	0.88 (0.70–1.16)	0.88 (0.62–1.36)	0.94 (0.81–1.11)	0.87 (0.72–1.06)	0.516
Total cholesterol (mmol/L)	3.64 ± 0.69	3.56 ± 0.90	3.95 ± 0.89	3.32 ± 0.64	**0.007**
LDL- cholesterol (mmol/L)	1.86 ± 0.55	1.85 ± 0.65	2.08 ± 0.73	1.74 ± 0.47	0.071
HDL-cholesterol (mmol/L)	1.33 ± 0.27	1.23 ± 0.40	1.37 ± 0.34	1.17 ± 0.28	0.086
Fasting glucose (mmol/L)	5.19 ± 0.73	5.09 ± 0.64	5.22 ± 0.82	5.08 ± 0.47	0.799
HOMA-IR^a^	1.60 (1.15–2.05)	1.22 (0.73–2.00)	1.48 (0.80–3.26)	1.36 (1.00–2.16)	0.724
Free T3(pmol/ml)^a^	12.57 (4.93–24.60)	17.21 (8.85–28.06)	22.57 (11.10–98.97)	29.51 (21.98–145.53)^‡^	**<0.001**
Free T4(pmol/ml)^a^	49.55 (28.83–81.47)	59.90 (37.11–76.59)	66.93 (45.54–86.68)^†^	69.01 (51.93–85.96)^‡^	**0.011**
TSH (mIU/L)^a^	0.00 (0.00–0.01)	0.01 (0.00–0.01)	0.01 (0.00–0.01)	0.01 (0.01–0.01)	0.338
TPOAb (IU/ml)^a^	19.95 (5.17–248.20)	54.22 (6.46–193.70)	9.06 (4.98–101.27)	16.81 (5.00–119.07)	0.492
TGAb (IU/mL)^a^	12.51 (2.49–58.88)	26.47 (7.72–182.10)	17.84 (10.21–112.26)	23.60 (14.43–150.87)^‡^	**0.050**
TRAb (IU/L)^a^	14.26 (1.55–24.31)	18.82 (6.82–27.48)	17.17 (10.31–23.74)	18.00 (9.03–29.81)^‡^	**0.015**

*BMI, body mass index; BP, blood pressure; LDL, low density lipoprotein; HDL, high density lipoprotein; HOMA-IR, homeostasis model assessment of insulin resistance; T3, triiodothyronine; T4, thyroxine; TSH, thyroid stimulating hormone; TPOAb, thyroid peroxide antibody; TGAb, thyroglobulin antibody; TRAb, thyrotropin receptor antibody; BMR, basal metabolic rate*.

*Data are presented as the mean ± SD or median (interquartile range)*.

§ *Adjusted for age and gender*.

† *P <0.05 compared with Q1 of serum Tsukushi*.

‡ *P <0.01 compared with Q1 of serum Tsukushi*.

a *Analysis performed on log-transformed data. Two-sided values of P <0.05 were considered statistically significant*.

**Figure 1:**
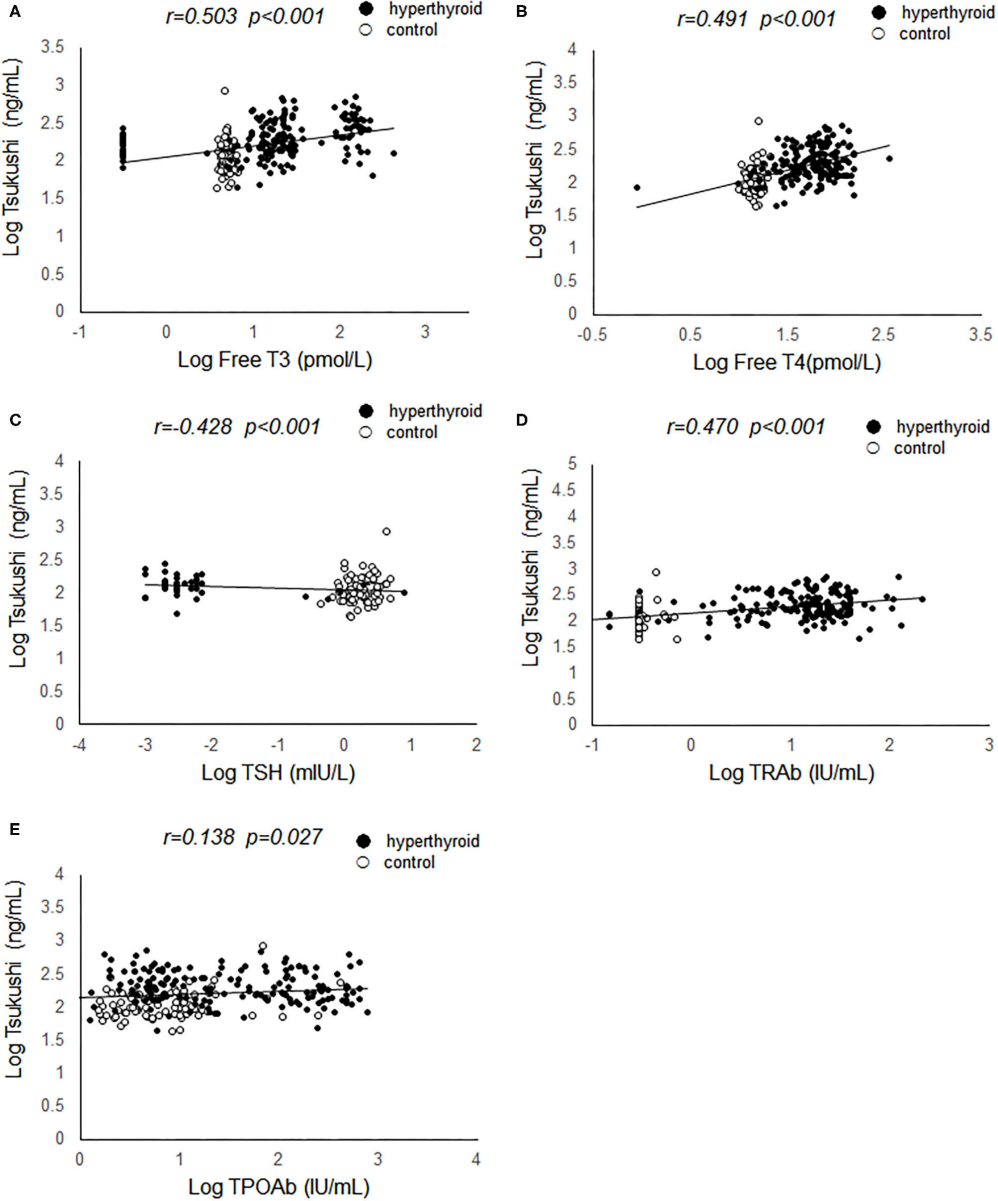
Correlation of serum Tsukushi levels with thyroid function. **(A)** Correlation of serum Tsukushi levels with serum levels of free T3; **(B)** correlation of serum Tsukushi levels with serum levels of free T4; **(C)** correlation of serum Tsukushi levels with serum levels of TSH; **(D)** correlation of serum Tsukushi levels with serum levels of TRAb; **(E)** correlation of serum Tsukushi levels with serum levels of TPOAb; T3, triiodothyronine; T4, thyroxine; TSH, thyroid stimulating hormone; TPOAb, thyroid peroxide antibody; TRAb, thyrotropin receptor antibody.

**Figure 2:**
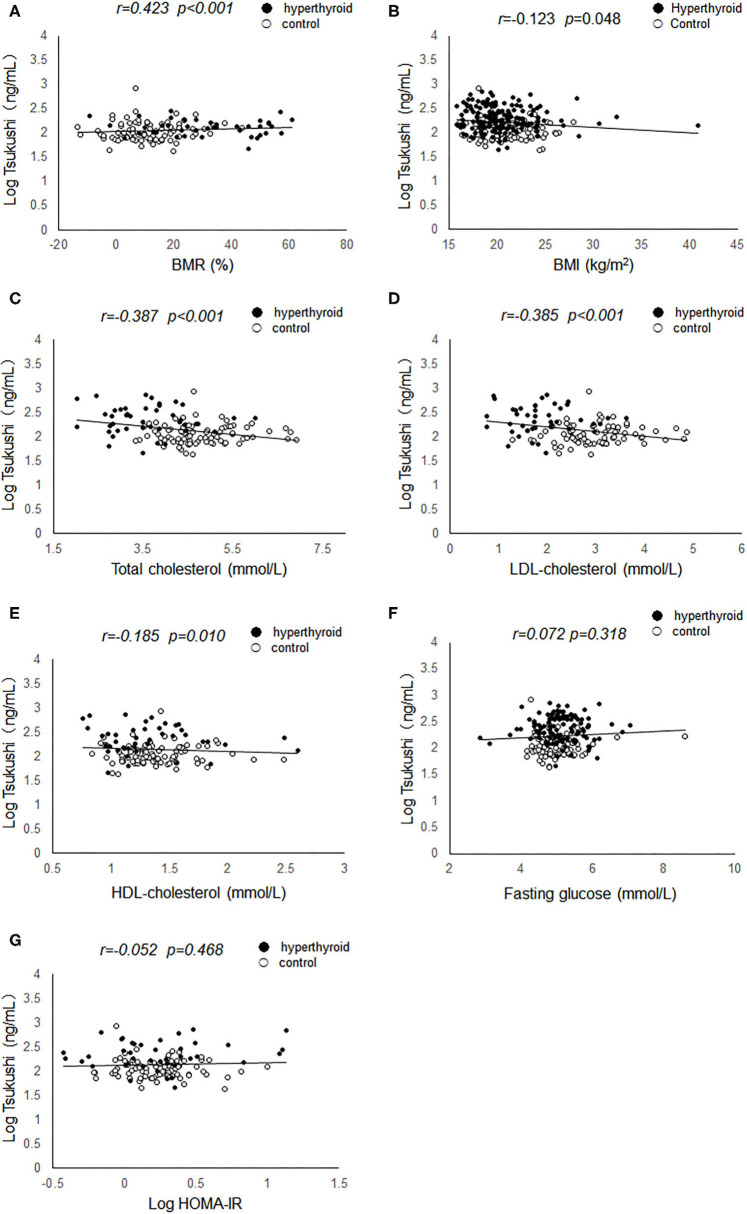
Correlation of serum Tsukushi levels with metabolic variables. **(A)** Correlation of serum Tsukushi levels with BMR; **(B)** correlation of serum Tsukushi levels with BMI; **(C)** correlation of serum Tsukushi levels with serum levels of total cholesterol; **(D)** correlation of serum Tsukushi levels with serum levels of LDL-cholesterol; **(E)** correlation of serum Tsukushi levels with serum levels of HDL-cholesterol; **(F)** correlation of serum Tsukushi levels with serum levels of fasting glucose; **(G)** correlation of serum Tsukushi levels with serum levels of HOMA-IR; BMR, basal metabolic rate; BMI, body mass index; LDL, low density lipoprotein; HDL, high density lipoprotein; HOMA-IR, homeostasis model assessment of insulin resistance.

Pearson's correlation analyses were used to examine the relationship between serum TSK levels and metabolic risk factors and thyroid hormones. As shown in Figure 1, serum TSK levels were positively correlated with free T3 (*r* = 0.503, *P* < 0.001) and free T4 (*r* = 0.491, *P* < 0.001), negatively correlated with serum TSH (*r* = −0.428, *P* < 0.001) in the hyperthyroid patients and healthy controls. Interestingly, serum TSK levels were also significantly correlated with thyroid antibodies, such as serum TRAb (*r* = 0.470, *P* < 0.001) and TPOAb (*r* = 0.138, *P* = 0.037). Additionally, serum TSK levels were positively correlated with BMR (*r* = 0.423, *P* < 0.001; Figure 2A) and negatively correlated with BMI (*r* = 0.123, *P* = 0.048; Figure 2B) and lipid profiles, including total cholesterol (*r* = 0.387, *P* < 0.001; Figure 2C), LDL-cholesterol (*r* = 0.385, *P* < 0.001; Figure 2D), and HDL-cholesterol (*r* = −0.185, *P* = 0.010; Figure 2E). Nevertheless, fasting glucose levels and HOMA-IR showed no significant correlation with serum TSK levels (Figures 2F,G).

Results of linear regression analyses of serum TSK level on metabolic risk factors and thyroid hormones were shown in Table 3. Serum TSK levels were significantly associated with thyroid hormones, including serum free T3, free T4 and TSH, and serum TRAb, adjusting for age, gender, smoking, and BMI. Likewise, Serum TSK levels were also significantly associated with metabolic risk factors, including total cholesterol, LDL-cholesterol, HDL-cholesterol; however, there were no significant associations of serum TSK levels with fasting glucose, systolic BP, diastolic BP, TPOAb, TGAb, and HOMA-IR.

**Table 3:** Clinical correlates of serum Tsukushi levels with clinical and biochemical variables.

**Variables**	**β**	**SE**	***P*-value**	**Multiple adjusted *P*-value^§^**
BMI (kg/m^2^)	−0.371	0.187	**0.048**	0.133
Waist circumference (cm)	−0.066	0.541	0.904	0.198
Systolic BP (mmHg)	1.492	0.897	0.098	0.096
Diastolic BP (mmHg)	−0.989	0.625	0.115	0.145
Total cholesterol (mmol/L)	−0.355	0.061	**<0.001**	**<0.001**
Total triglycerides (mmol/L)^a^	0.009	0.012	0.418	0.311
LDL-cholesterol (mmol/L)	−0.301	0.052	**<0.001**	**<0.001**
HDL-cholesterol (mmol/L)	−0.055	0.021	**0.010**	**0.004**
Fasting glucose (mmol/L)	0.042	0.042	0.318	0.500
HOMA-IR^a^	−0.015	0.021	0.468	0.775
Free T3 (pmol/ml)^a^	0.263	0.028	**<0.001**	**<0.001**
Free T4 (pmol/ml)^a^	0.169	0.019	**<0.001**	**<0.001**
TSH (mIU/L)^a^	−0.542	0.072	**<0.001**	**<0.001**
TPOAb (IU/ml)^a^	0.109	0.049	**0.027**	0.074
TGAb (IU/mL)^a^	0.067	0.050	0.183	0.372
TRAb (IU/L)^a^	0.412	0.048	**<0.001**	**<0.001**
BMR (%)	10.420	1.410	**<0.001**	**<0.001**

*BMI, body mass index; BP, blood pressure; LDL, low density lipoprotein; HDL, high density lipoprotein; HOMA-IR, homeostasis model assessment of insulin resistance; T3, triiodothyronine; T4, thyroxine; TSH, thyroid stimulating hormone; TPOAb, thyroid peroxide antibody; TGAb, thyroglobulin antibody; TRAb, thyrotropin receptor antibody; BMR, basal metabolic rate*.

§ *Adjusted for age, gender, smoking, and BMI*.

a *Analysis performed on log-transformed data. Two-sided values of P <0.05 were considered statistically significant*.

The multivariable-adjusted odds ratios (ORs) for the association of serum TSK levels and metabolic risk factors with hyperthyroidism are shown in Table 4. Subjects in the lowest quartile of serum TSK levels had significantly lower risks of hyperthyroidism than those in higher quartiles (all *P* < 0.001), adjusting for age and gender. Likewise, serum TSK levels were significantly associated with risks of hyperthyroidism, after adjusting for age and gender [OR (95% CI), 1.014 (1.009–1.019); *P* < 0.001]. Of note, the ORs for hyperthyroidism remained significant, even after adjusting for BMI, fasting glucose, HOMA-IR, and LDL-cholesterol [OR (95% CI), 1.012 (1.005–1.019); *P* < 0.001].

**Table 4:** Odds ratios of hyperthyroidism according to serum Tsukushi levels.

	**OR**	**95% CI**	***P*-value**
**Model 1**			
Serum Tsukushi (ng/ml)	1.014	1.009–1.019	**<0.001**
Serum Tsukushi (ng/ml)			
(Quartile 2 vs. Quartile 1)	1.032	1.017–1.047	**<0.001**
(Quartile 3 vs. Quartile 1)	1.022	1.014–1.030	**<0.001**
(Quartile 4 vs. Quartile 1)	1.012	1.008–1.017	**<0.001**
**Model 2**			
Serum Tsukushi (ng/ml)	1.014	1.009–1.019	**<0.001**
Serum Tsukushi (ng/ml)			
(Quartile 2 vs. Quartile 1)	1.031	1.017–1.046	**<0.001**
(Quartile 3 vs. Quartile 1)	1.022	1.014–1.030	**<0.001**
(Quartile 4 vs. Quartile 1)	1.012	1.007–1.016	**<0.001**
**Model 3**			
BMI (kg/m^2^)	0.856	0.701–1.046	0.129
Fasting glucose (mmol/L)	1.918	0.839–4.387	0.123
HOMA-IR	0.815	0.608–1.091	0.170
LDL-cholesterol(mmol/L)	0.063	0.025–0.159	**<0.001**
Serum Tsukushi (ng/ml)	1.012	1.005–1.019	**0.001**
Serum Tsukushi (ng/ml)			
(Quartile 2 vs. Quartile 1)	1.043	1.010–1.078	**0.011**
(Quartile 3 vs. Quartile 1)	1.028	1.013–1.044	**<0.001**
(Quartile 4 vs. Quartile 1)	1.009	1.004–1.015	**0.002**

*OR, odds ratio; CI, confidence interval; BMI, body mass index; HOMA-IR, homeostasis model assessment of insulin resistance; LDL, low density lipoprotein*.

*Model 1: without adjustment*.

*Model 2: adjusted for age, gender*.

*Model 3: adjusted for model 2 + BMI, fasting glucose, HOMA-IR, and LDL-cholesterol. Two-sided values of P <0.05 were considered statistically significant*.

### Circulating TSK Levels After Thionamide Treatment

The change of circulating TSK levels and thyroid hormones after thionamide treatment were shown in Table 5. Serum free T3 and free T4 concentrations reached to normal levels after 3-months thionamide treatment; meanwhile, serum TSK levels were dramatically decreased from 188.52 (138.73–361.41) ng/mL to 93.01 (73.80–120.68) ng/mL (*P* < 0.001). Additionally, BMR, heart rate, ALT, AST, and metabolic risk factors, including BMI, waist circumference, total cholesterol, LDL-cholesterol, and HDL-cholesterol, were significantly improved after thionamide treatment.

**Table 5:** Effect of thionamide treatment on serum Tsukushi levels in hyperthyroid patients.

**Variables**	**Before**	**After**	***P*-value**
Sample size	46	46	
Age (years)	31.6 ± 11.1	–	–
Gender (female *n*, %)	34 (73.9)	–	–
Current smokers (*n*, %)	4 (8.5)	3 (6.4)	**<0.001**
BMI (kg/m^2^)	20.3 ± 2.8	21.4 ± 2.7	**<0.001**
Waist circumference (cm)	75.0 ± 8.5	77.2 ± 7.06	**<0.001**
Systolic BP (mmHg)	123.4 ± 15.8	116.6 ± 14.0	**0.001**
Diastolic BP (mmHg)	72.7 ± 11.7	73.9 ± 9.8	0.511
Heart rate (beats/minute)	104.0 ± 11.4	82.1 ± 8.0	**<0.001**
Triglycerides (mmol/L)^a^	0.87 (0.71–1.06)	1.05 (0.74–1.33)	0.130
Total cholesterol (mmol/L)	3.72 ± 0.84	5.40 ± 0.99	**<0.001**
LDL- cholesterol (mmol/L)	1.88 ± 0.57	3.26 ± 0.77	**<0.001**
HDL-cholesterol (mmol/L)	1.42 ± 0.42	1.62 ± 0.33	**0.001**
Fasting glucose (mmol/L)	5.26 ± 0.63	5.03 ± 0.44	**0.013**
HOMA-IR^a^	1.63 (1.13–2.57)	1.68 (1.14–1.93)	0.290
ALT (U/L)	36.8 ± 15.0	22.3 ± 13.0	**<0.001**
AST (U/L)	28.6 ± 11.3	20.9 ± 7.2	**<0.001**
Free T3 (pmol/L)^a^	21.23 (14.60–100.23)	4.58 (3.83–5.40)	**<0.001**
Free T4 (pmol/L)^a^	67.16 (40.15–82.01)	13.05 (11.60–15.39)	**<0.001**
Total T3 (nmol/L)^a^	7.63 (5.29–12.32)	1.55 (1.28–2.15)	**<0.001**
Total T4 (nmol/L)^a^	379.00 (268.80–387.00)	94.60 (79.75–110.95)	**<0.001**
TSH (mIU/L)^a^	0.01 (0.00–0.01)	0.49 (0.01–3.66)	**<0.001**
TPOAb (IU/ml)^a^	26.70 (5.68–127.20)	52.17 (5.84–299.15)	0.253
TGAb (IU/mL)^a^	32.69 (10.21–243.08)	22.31 (12.99–152.46)	0.949
TRAb (IU/L)^a^	17.39 (7.64–28.28)	8.40 (2.24–17.69)	**<0.001**
BMR (%)	43.32 ± 14.33	13.78 ± 11.74	**<0.001**
Serum Tsukushi (ng/ml)^a^	188.52 (138.73–361.41)	93.01 (73.80–120.68)	**<0.001**

*BMI, body mass index; BP, blood pressure; LDL, low density lipoprotein; HDL, high density lipoprotein; HOMA-IR, homeostasis model assessment of insulin resistance; ALT, alanine transaminase; AST, aspartate aminotransferase; T3, triiodothyronine; T4, thyroxine; TSH, thyroid stimulating hormone; TPOAb, thyroid peroxide antibody; TGAb, thyroglobulin antibody; TRAb, thyrotropin receptor antibody; BMR, basal metabolic rate*.

*Data are presented as the mean ± SD or median (interquartile range)*.

a *Analysis performed on log-transformed data. Two-sided values of P <0.05 were considered statistically significant*.

## Discussion

In the present study, we provided, for the first time, evidence that circulating TSK concentrations were markedly elevated in patients with hyperthyroidism and dramatically declined with improvement of thyroid function. Furthermore, increased circulating TSK concentrations were independently associated with hyperthyroidism. This study provides strong evidence that circulating TSK plays as a prognostic factor for hypermetabolic status in relation to hyperthyroidism. These findings have important clinical implications.

It has been well-documented that thyroid hormones are involved in the regulation of metabolism as well as thermogenesis; meanwhile, thyroid hormone status correlates with body weight and energy expenditure (6, 23, 24). Thyroid hormone directly stimulates thermogenesis in brown adipose tissue and also induces white adipose tissue browning through peripheral and central mechanisms (6, 25–27). Recent studies have shown that altered thyroid status can affect circulating levels of several cytokines secreted from the liver, which may be involved in the thyroid hormone signaling network to modulate whole-body glucose and lipid homeostasis (13, 15, 28, 29). Wang et al. identified TSK as a liver-enriched secreted factor that was remarkably induced by T3 treatment or cold exposure in animal models, and was tightly linked to the activation of thermogenesis and energy expenditure (20–22). Unfortunately, information regarding the association of circulating TSK with thyroid function and energy expenditure in humans was limited.

In the present study, our data indicated that serum TSK levels are significantly elevated in patients with hyperthyroidism compared to healthy controls. Meanwhile, the reversal of the hyperthyroid state was followed by the decline of serum TSK levels in patients with hyperthyroidism. Of note, circulating TSK concentrations were positively associated with serum free T3 and free T4 levels. Furthermore, we noted in this study that circulating TSK concentrations were significantly associated with serum thyroid antibodies levels including serum TRAb and TPOAb. To date, this study is the first clinical study to determine the role of circulating TSK in the development of hyperthyroidism in humans. These findings suggest that circulating TSK concentrations have prognostic significance in patients with hyperthyroidism. However, these observations should be confirmed and replicated in other population studies.

It has been well-established that excess thyroid hormone promotes a hypermetabolic state characterized by increased energy expenditure, weight loss, reduced cholesterol levels, and increased gluconeogenesis (1, 3). Previous studies suggest that TSK acts as a hormonal checkpoint that suppresses adipose tissue sympathetic innervation, adrenergic action and thermogenesis, and attenuates energy expenditure (20). In animal models, TSK deficiency protected mice from high-fat diet induced obesity (20, 22). In contrast, Mouchiroud et al. reported that TSK did not affect brown fat thermogenic capacity or body weight gain in TSK knockout mice model (30). Our data demonstrated that serum TSK levels were positively associated with BMR in hyperthyroid patients and negatively correlated with BMI, indicating that TSK may be involved in activation of thermogenesis and energy expenditure in hyperthyroidism.

Additionally, our data indicated that serum TSK levels were negatively correlated with serum cholesterol levels in patients with hyperthyroidism, including levels of total cholesterol, LDL-cholesterol and HDL-cholesterol. It has been well-established that thyroid hormones majorly stimulates transcription of the low-density lipoprotein receptor (LDL-R) gene stimulating uptake of cholesterol and enhanced cholesterol synthesis (31). Thyroid hormone regulates cholesterol and bile acid homeostasis through direct induction of sterol response element binding protein 2 (SREBP-2) and CPY7A1 gene expression as well as cross-talk with other nuclear receptors, such as peroxisome proliferator-activated receptor α (PPARα) and liver X receptor α pathway (8, 9, 32–34). Similarly, studies indicated that TSK affect cholesterol homeostasis by modulating circulating HDL-cholesterol, reversing cholesterol transport, and bile acids synthesis in the liver (22). Furthermore, previous study also reported that TSK deficiency can significantly improve insulin sensitivity and glucose tolerance in animal model (20). However, our data did not find a significant association of circulating TSK concentrations with fasting glucose and insulin resistance. Altogether, these findings suggest that elevated TSK concentrations in patients with hyperthyroidism may in part mediate thyroid hormone action on energy homeostasis and lipid metabolism. Prospective cohort studies are needed to confirm this finding and elucidate potential metabolic mechanisms connecting TSK and thyroid hormones.

This case-control study provided an opportunity to investigate the role of circulating TSK in hyperthyroidism. Nevertheless, there are several limitations in the current study. First, the study was based on case-control data that was recruited from single medical center with a relatively limited sample size. Further studies should replicate and determine the role of circulating TSK in other population studies. Second, given its cross-sectional design, it is not possible determine the causal relationship between serum T3 and TSK levels in the development of hyperthyroidism. Therefore, the causal association between circulating TSK and hyperthyroidism should be further studied *in vivo* and *in vitro*, and evaluated in prospective cohort studies with larger sample sizes and longer follow-up periods.

In conclusion, our study provides clinical evidence for the first time revealing that circulating TSK concentrations were markedly elevated in patients with hyperthyroidism and independently associated with risks of hyperthyroidism in relation to hypermetabolic state. These findings indicate that circulating TSK may be involved in the thyroid hormone signaling network to modulate energy homeostasis and lipid metabolism, and the development of hyperthyroidism in humans. However, the role of circulating TSK in the pathological process of hyperthyroidism needs to be further studied *in vivo* and *in vitro*.

## Data Availability Statement

The raw data supporting the conclusions of this article will be made available by the authors, without undue reservation.

## Ethics Statement

The studies involving human participants were reviewed and approved by the Institutional Review Board of Nanfang Hospital of Southern Medical University. The patients/participants provided their written informed consent to participate in this study.

## Author's Note

Hyperthyroidism is characterized by hypermetabolic state such as weight loss, increased energy expenditure, accelerated lipolysis and hepatic gluconeogenesis. It occurs due to excessive thyroid hormone synthesis and secretion from the thyroid gland. Recent advances suggest that the liver may involve in the thyroid hormone signaling network to modulate whole-body glucose and lipid homeostasis through the secretion of hepatokines. It is important to investigate the role of hepatokines in crosstalk with thyroid hormone signaling pathway in pathogenesis of hyperthyroidism. There are observations on metabolic crosstalk of Tsukushi (TSK) with thyroid hormone signal tightly linked to the activation of thermogenesis and energy expenditure in animal models. However, information is not available regarding the association between circulating TSK concentrations and hyperthyroidism in humans. Our study provides clinical evidence for the first time revealing that circulating TSK concentrations were markedly elevated in patients with hyperthyroidism and independently associated with risks of hyperthyroidism in relation to hypermetabolic state. Our findings indicate that circulating TSK may be involved in the thyroid hormone signaling network to modulate energy homeostasis and lipid metabolism, and act as a predictive factor in the development of hyperthyroidism in humans.

## Author Contributions

All authors listed have made a substantial, direct and intellectual contribution to the work, and approved it for publication.

## Conflict of Interest

The authors declare that the research was conducted in the absence of any commercial or financial relationships that could be construed as a potential conflict of interest.
